# Magnetic resonance fingerprinting of the pancreas at 1.5 T and 3.0 T

**DOI:** 10.1038/s41598-020-74462-6

**Published:** 2020-10-16

**Authors:** Eva M. Serrao, Dimitri A. Kessler, Bruno Carmo, Lucian Beer, Kevin M. Brindle, Guido Buonincontri, Ferdia A. Gallagher, Fiona J. Gilbert, Edmund Godfrey, Martin J. Graves, Mary A. McLean, Evis Sala, Rolf F. Schulte, Joshua D. Kaggie

**Affiliations:** 1 Department of Radiology, University of Cambridge, Cambridge Biomedical Campus, Box 218, Cambridge, CB2 0QQ UK; 2 Addenbrooke’s Hospital, Cambridge University Hospitals NHS Foundation Trust, Cambridge, UK; 3 Cancer Research UK, Cambridge, UK; 4 IMAGO7 Foundation, Pisa, Italy; 5 GE Healthcare, Munich, Germany

**Keywords:** Diagnostic markers, Medical imaging

## Abstract

Magnetic resonance imaging of the pancreas is increasingly used as an important diagnostic modality for characterisation of pancreatic lesions. Pancreatic MRI protocols are mostly qualitative due to time constraints and motion sensitivity. MR Fingerprinting is an innovative acquisition technique that provides qualitative data and quantitative parameter maps from a single free‐breathing acquisition with the potential to reduce exam times. This work investigates the feasibility of MRF parameter mapping for pancreatic imaging in the presence of free-breathing exam. Sixteen healthy participants were prospectively imaged using MRF framework. Regions-of-interest were drawn in multiple solid organs including the pancreas and T_1_ and T_2_ values determined. MRF T_1_ and T_2_ mapping was performed successfully in all participants (acquisition time:2.4–3.6 min). Mean pancreatic T_1_ values were 37–43% lower than those of the muscle, spleen, and kidney at both 1.5 and 3.0 T. For these organs, the mean pancreatic T_2_ values were nearly 40% at 1.5 T and < 12% at 3.0 T. The feasibility of MRF at 1.5 T and 3 T was demonstrated in the pancreas. By enabling fast and free-breathing quantitation, MRF has the potential to add value during the clinical characterisation and grading of pathological conditions, such as pancreatitis or cancer.

## Introduction

Magnetic resonance imaging (MRI) of the pancreas is increasingly used as a major diagnostic modality for characterisation of pancreatic lesions, given its superior soft tissue contrast and increased sensitivity for detection and characterisation of smaller pancreatic masses^1,2^. However, the wider use of MRI remains hampered by long examination times, which limits the types of acquisition to the minimum required for basic diagnosis. Contrast-enhanced CT is routinely used in the context of pancreatic disease given its wide availability and acquisition time. However, radiation exposure and iodine allergy are its major risks. Endoscopic ultrasonography (EUS) is the most sensible imaging method for the pancreas^3,4^, but it is invasive, operator dependent and associated with complications.

T_1_ and T_2_ relaxation times are valuable quantitative parameters to characterize different tissues, particularly in the assessment of myocardial and liver diseases^5–9^. Thus far only some studies have reported on the value of quantitative MRI on the pancreas. In normal pancreas, T_1_ values have been reported to reflect the amount of acinar protein, rough endoplasmic reticulum and fat infiltration^10–13^. T_1_ maps were found to aid determining the presence and severity of acinar cell loss in the diagnosis and classification of chronic pancreatitis^10,11,14^. Multiparametric MRI comprising T1, T2, and ADC mapping was also shown useful in discriminating different pancreatic processes^15^. Despite the potential of quantitative multiparametric MRI, pancreatic MRI protocols are still mostly qualitative, with clinical assessments involving a trained reader to create a subjective evaluation based on T_1_- and T_2_-weighted images. This subjective evaluation is highly parameter dependent, which reduces the ability for analysis to be translated across centres. Quantitative analysis is mostly undertaken in a research context, often limited to conventional methods, due to the significant scan time required and technical limitations such as field inhomogeneities and patient motion^9,16^.

Several quantitative methods have been described for fast abdominal imaging^17–19^. MR fingerprinting (MRF) is an innovative technique that provides qualitative and quantitative data from a single exam^19^. As a T_1_ and T_2_ mapping method, MRF has demonstrated itself as a fast, repeatable within a system and reproducible across centres^20^. MRF has been shown to be quite insensitive to motion due to incoherent sampling resulting from the golden angle rotations, which enable pattern matching if a voxel is static for sufficient frames^21^. MRF-derived multiparametric maps have shown diagnostic utility in the brain^22–24^ and abdomen^25–27^. In tumours, MRF measurements have shown T_1_ values in lesions that are nearly double those of normal-appearing tissue in the prostate, liver and brain, and demonstrated T_2_ differences between low and high grade tumours as great as 70%^24–27^. The application of MRF in the pancreas, and in the abdomen in general, offers a wide range of clinical and research opportunities by accelerating acquisition of quantitative parameter maps.

The challenges associated with MRF are similar to those for any quantitative MRI technique: motion, spatial resolution, and B_0_ and B_1_ field non-uniformity. MRF may also be sensitive to magnetization transfer, partial volume, and slice profile effects^21,28–32^. Spiral sampling use in MRF is advantageous in that it may reduce motion sensitivity by oversampling the centre of k-space^33^ and matching a temporal pattern with MRF reconstruction.

This work investigates the feasibility of MRF for pancreatic imaging in the presence of free-breathing motion for the first time. Pancreatic multiparametric MRI has shown potential clinical impact in the assessment of pancreatic disease. We predict that MRF acquisition might be of particular value in pancreatic cancer (PCa) patients, who are often frail and therefore less compliant, allowing for improved quantitative clinical assessment to be performed. Most MRF investigations published to date have been performed at 3.0 T; however, the use of MRF at 1.5 T would increase its clinical potential given the wider availability and clinical indications for these systems. Here we compare the performance of MRF in the abdomen at both 1.5 T and 3.0 T in healthy subjects.

## Results

### Feasibility of MRF of the pancreas at 1.5 T and 3 T

MRF T_1_ and T_2_ maps were obtained for each healthy volunteer. The mean age for the 16 participants was 33 years, of which 6 (40%) were female. Sixteen participants (100%) underwent MRI at 3.0 T and 12 (80%) underwent MRI at 1.5 T. The volunteers had normal appearances of the liver, pancreas, kidneys and spleen as assessed by a radiologist. The MRF acquisition time was 146–215 s (2.4–3.6 min) per subject. Dictionary simulation normally required approximately 45 min for reconstruction with parallelized code. The reconstruction of each channel, slice, and frame was the largest time-consuming step prior to compression of the dataset with singular value decomposition (SVD) factorization. After SVD compression, the T_1_ and T_2_ maps were determined by finding the maximum inner product between the simulated signal and the acquired images. Product pattern matching took a few seconds. The abdominal images required 3.5 h for non-Cartesian reconstruction and matching due to the large number of reconstructed frames and coil channels.

### Quantitative and qualitative interpretation of the MRF maps

**Figure 1:**
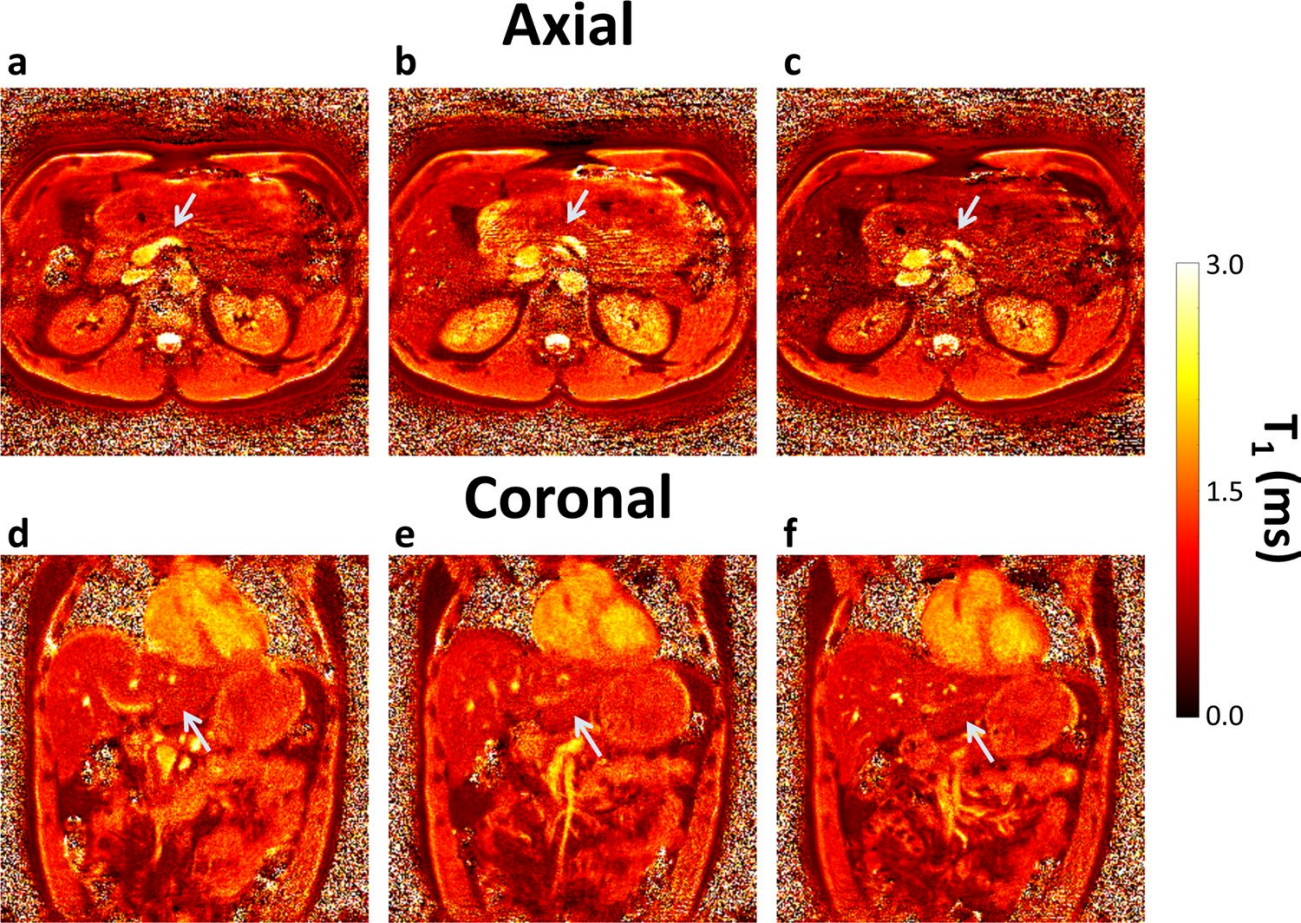
Three consecutive slices showing free breathing T_1_ maps obtained with 2D MRF, axially (**a–c**) and coronally (**d–f**). The same flip angle list, gradient trajectory, and reconstruction was used. The axial images (**a–c**) resulted in inconsistent values between slices, as observed in the kidneys, as well as the appearance of aliasing artefacts, despite a sufficiently large field-of-view. These artefacts are not present in the coronal images (**d–f**). The pancreas is noted with a blue arrow in the images.

Axial and coronal images were acquired at 3.0 T in the same subject. Coronal acquisition was found to be more optimal to image the upper abdomen (pancreas), avoiding most of the artefacts encountered with axial imaging (Fig. 1). These artefacts, created primarily due to motion but also undersampling, caused underestimation of the T_1_ means for each single subject, in example the T_1_ mean value of the whole kidney reduced from 2071 ± 428 ms (coronal) to 1045 ± 369 ms (axial). In the pancreas the T_1_ means was up to 100 ms higher in the coronal plane.

**Figure 2:**
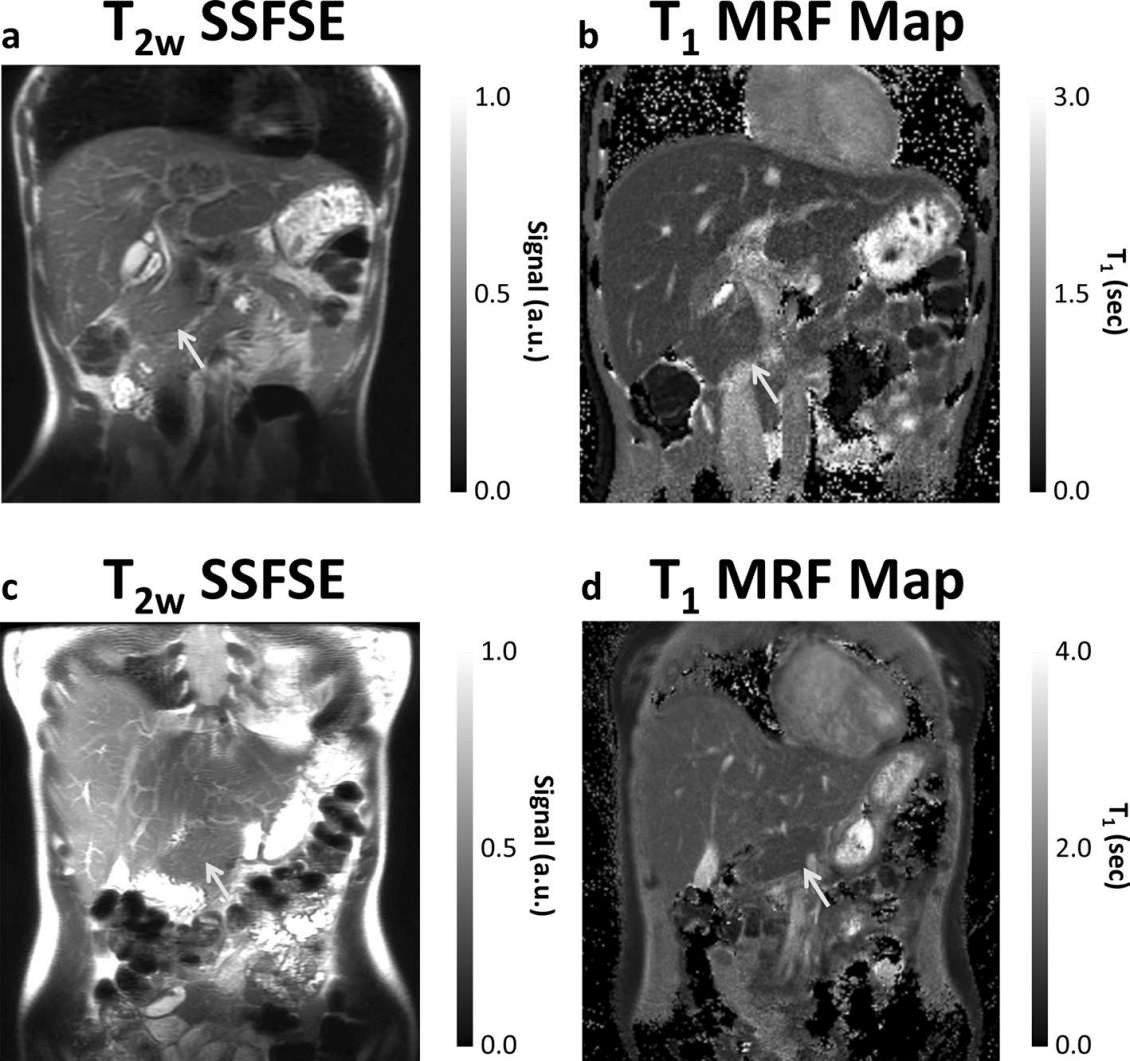
(**a**, **c**) T_2_-weighted SSFSE images and (**b**, **d**) MRF-derived T_1_ maps of the abdomen at (**a**, **b**) 1.5 T and (**c**, **d**) 3.0 T. This demonstrates the good anatomic detail and homogeneous signal throughout the pancreatic gland, obtained by MRF maps when compared with the conventional SSFSE image. The liver and pancreas (arrowed) have similar intensities in the T_1_ maps, while fat, with a much shorter T_1_, appears dark and muscle, with a longer T_1_ appears bright. SSFSE: single-shot-fast-spin-echo sequence, MRF: magnetic resonance fingerprinting.

**Figure 3:**
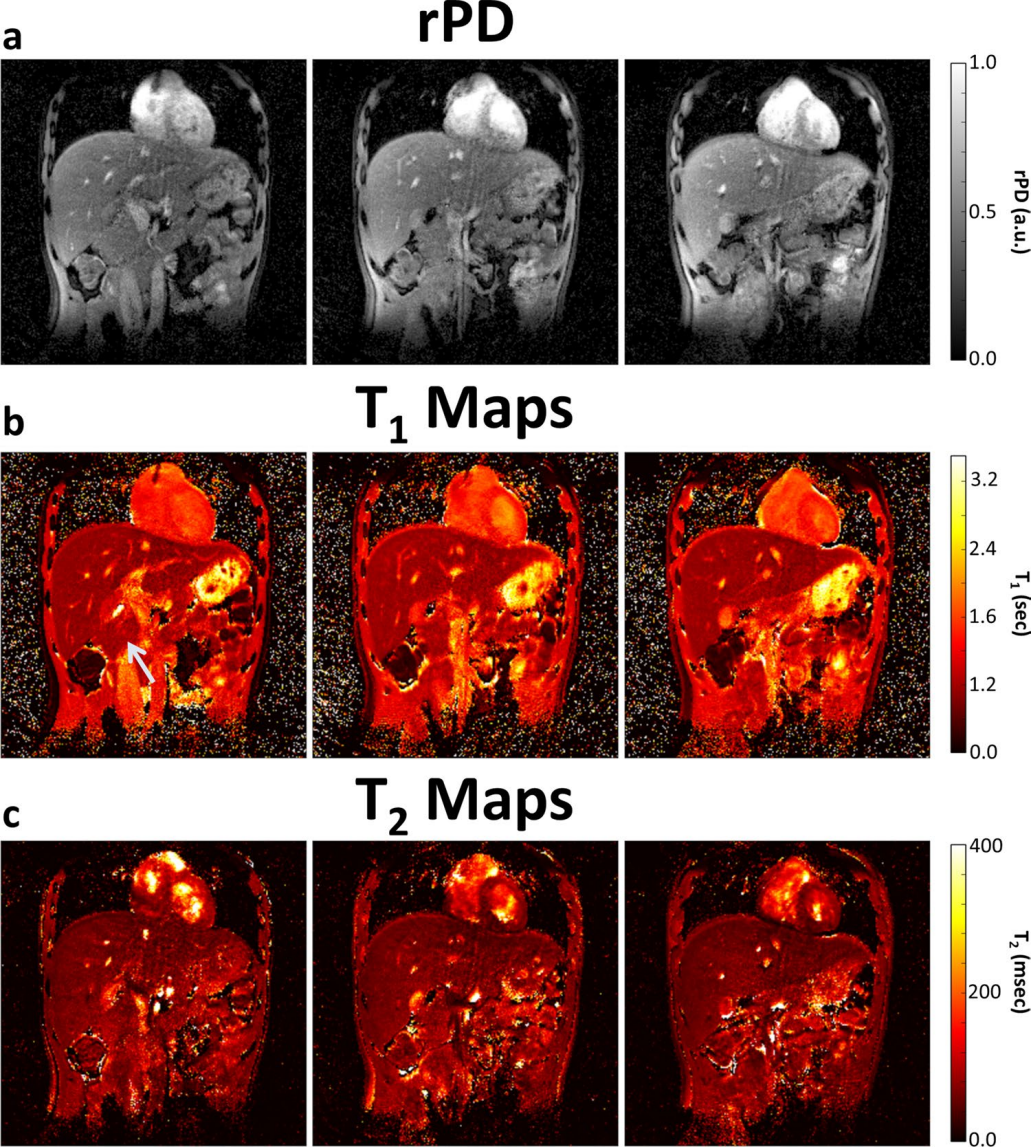
MRF-derived maps of **(a**) relative proton density (rPD), (**b**) T_1_, and (**c**) T_2_ of three consecutive slices (posterior to anterior) within the abdomen at 1.5 T. These images show the ability of MRF to obtain multiple slices through the abdomen with reasonable anatomical detail and low motion artefact. The pancreas (arrowed) has homogeneous signal throughout in both T_1_ and T_2_ maps, and is distinguishable due to fat/water boundaries at its periphery.

**Figure 4:**
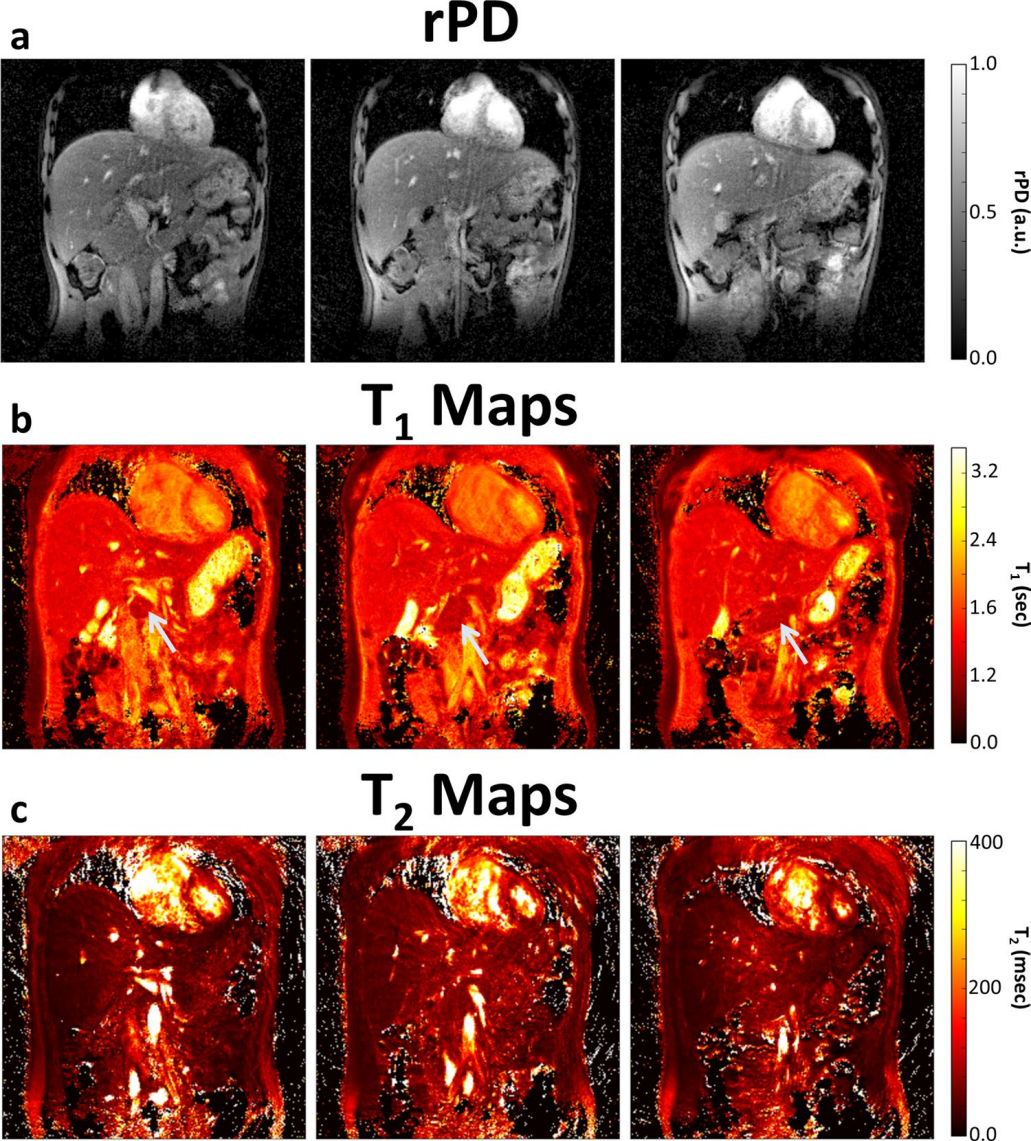
(**a**) Relative proton density (rPD), (**b**) T_1_ maps, and (**c**) T_2_ maps of three consecutive slices (posterior to anterior) within the abdomen at 3.0 T. These images show the ability of MRF to obtain multiple slices through the abdomen with reasonable anatomical detail and low motion artefact. Again, homogeneous signal was shown throughout the pancreas (arrowed). These T_2_ maps result in larger spiral artefacts that are not present on the T_1_ maps or at 1.5 T.

**Figure 5:**
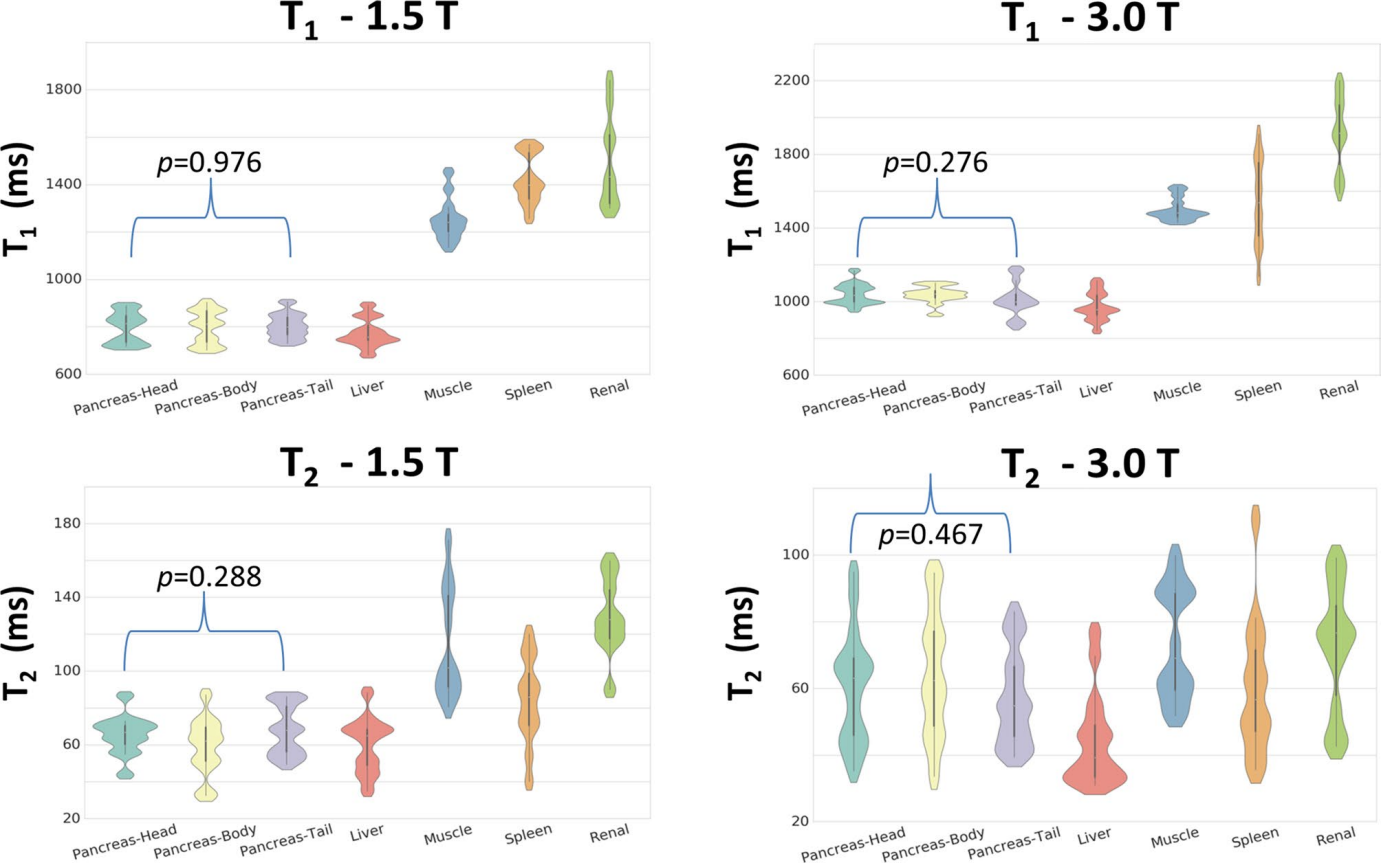
Tissue and field specific T_1_ and T_2_ distributions. Similar T_1_ and T_2_ patterns are visible at 1.5 and 3.0 T. The differences between the mean value of the multiple tissues are greater for T_2_ at 1.5 T, and for T_1_ at 3.0 T. No significant differences were found in the T_1_ and T_2_ values, at both magnetic field strengths, between the pancreas head, body and tail, according to one-way ANOVA (*p* > 0.05).

T_2_-weighted single-shot-fast-spin-echo sequence (SSFSE) images and MRF T_1_ maps are shown in Fig. 2. These images highlight the good anatomic detail obtained by MRF maps when compared with the conventional SSFSE image. The pancreatic gland showed homogeneous signal throughout the gland in all volunteers (Figs. 2, 3, 4 and 5 and Table 1), with similar contrast (i.e., T_1_ and T_2_) found in the liver and pancreas, regardless of imaging method or field strength. Three slices of representative relative proton density (rPD), T_1_ and T_2_ maps are shown in Figs. 3 and 4.

**Table 1:** Mean and standard deviation of T_1_ and T_2_ values for each pancreatic region. The references are: γ^34^, ‡^16^,*^14^, †^9^, φ^35^, &^15^.

	1.5 T				3.0 T			
	T_1_ (ms)		T_2_ (ms)		T_1_ (ms)		T_2_ (ms)	
	MRF	Lit. γ	MRF	Lit. ‡	MRF	Lit.	MRF	Lit. †,^φ^ MRF Lit. ^&^
Pancreas head	798 (67)		65 (10)	61 (7)	1041 (58)	844 (216)* 846 (74)^φ^	61 (17)	60 (8)† 47 (3)^φ^
Pancreas body	799 (72)		59 (16)	59 (5)	1038 (46)	884 (242)* 854 (85)^φ^	65 (19)	64 (12)† 48 (4)^φ^
Pancreas tail	803 (53)		68 (14)	59 (3)	1010 (92)	866 (266)* 870 (83)^φ^	57 (14)	67 (16)† 47^φ^
Average		584 (14)			1029 (65)	863 (90)^&^	61 (17)	33 (4)^&^

Tissue-specific T_1_ and T_2_ values, at 1.5 T and 3 T, are reported in Tables 1 and 2 (pancreas and non-pancreas, respectively) and Fig. 5. The pancreas (head, body, and tail) showed relatively homogenous appearances and T_1_ and T_2_ values, which are listed in Table 1. No significant differences in T_1_ and T_2_ values were found between the pancreatic head, body, and tail (*p* > 0.05).

**Table 2:** Mean and standard deviation of T_1_ and T_2_ values of non-pancreatic abdominal regions. All measured regions had T_1_ values 8–28% longer and T_2_ values 36–80% shorter at 3.0 T when compared with 1.5 T. The reference for γ is ^34^ and ‡ is ^25^.

	1.5 T				3.0 T					
	T_1_ (ms)		T_2_ (ms)		T_1_ (ms)			T_2_ (ms)		
	MRF	Lit.^γ^	MRF	Lit. ^γ^	MRF	Lit. ^γ^	MRF Lit.^‡^	MRF	Lit.^γ^	MRF Lit.^‡^
Liver	774 (62)	586 (39)	60 (15)	46 (6)	974 (78)	809 (71)	745 (65)	44 (14)	34 (4)	31 (6)
Muscle	1253 (92)	856 (61)	115 (30)	27 (8)	1500 (63)	898 (33)	1100 (59)	74 (17)	29 (4)	44 (9)
Spleen	1420 (105)	1057 (42)	84 (24)	79 (15)	1544 (237)	1328 (31)	1232 (92)	60 (20)	61 (9)	60 (19)
Kidney	1503 (200)	1189 (58)	129 (21)	86 (7)	1911 (24)	1343 (148)	1702 (205)	72 (19)	79 (8)	60 (21)

As expected, T_1_ relaxation times for all organs were significantly longer at 3 T (1.5 T 1.02 ± 0.3 s vs. 3.0 T 1.26 ± 0.38 s, *p* = 0.0001). Similar T_1_ and T_2_ trends between organs were observed at both field strengths, although the trends were more pronounced for both T_1_ and T_2_ at 1.5 T and for T_1_ at 3.0 T. The liver and pancreas could not be differentiated based on the T_1_ or T_2_ relaxation values at both field strengths.

The pancreatic T_1_ values were 30–50% lower than in muscle, spleen, and kidney at both 1.5 and 3.0 T. Large standard deviations were found for the spleen, muscle and kidney. However, in the case of the kidney the drawn ROI included both medulla and cortex, which have different relaxation times^25^.

### Phantom results

**Figure 6:**
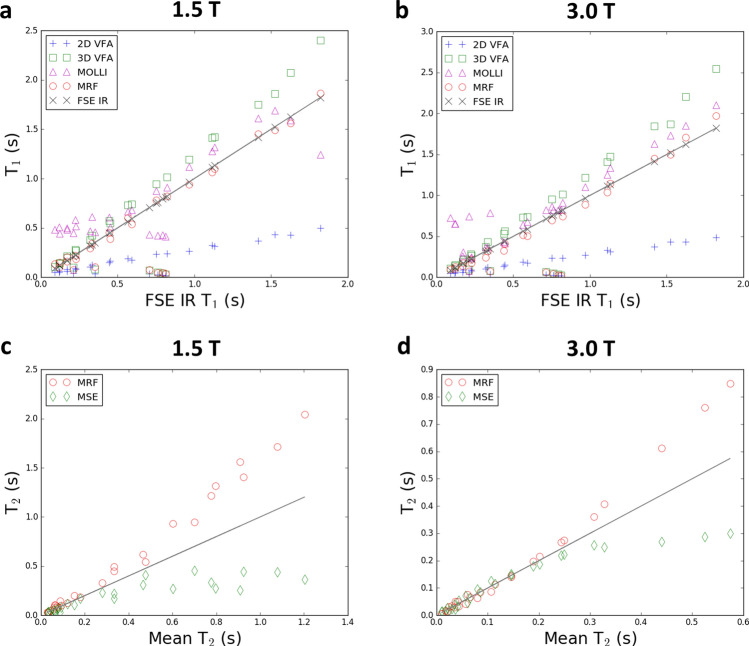
T_1_ and T_2_ phantom values at both 1.5 T and 3.0 T, using conventional techniques and MRF. T_1_ values between MRF and FSE IR had low absolute mean differences (< 0.05 s for all vials above 0.1 s). There were four vials where the 2D VFA, 3D VFA, and 2D MRF all failed, which occurred at the centre of the T_2_-layer of the NIST phantom where the T_2_ values were < 30 ms. The MSE flattened at longer T_2_ values. MOLLI-T_1_ and 3D VFA was higher than both MRF-T_1_ and FSE-IR-T_1_.

The phantom results are shown in Fig. 6, demonstrating the results of several T_1_ and T_2_ mapping methods.

For T_1_ mapping, these show that MRF agrees with fast spin echo with inversion recovery (FSE-IR) values, which is considered close to gold standard. The 2D variable flip angle (VFA) method vastly underestimated T_1_, whereas the 3D VFA method overestimated T_1_. The modified Look-Locker inversion recovery (MOLLI) approach overestimated T_1_ mildly, when compared with FSE-IR above 500 ms; below 500 ms, the MOLLI-T_1_ values plateaued. These biases were similar between both 1.5 T and 3.0 T results. As a quantitative metric of agreement, the sum across all 28 vials of the absolute differences, and divided by 28 to account for additional samples, between the T_1_ methods and the FSE-IR results were: for 3.0 T, 0.16 s for MRF, 0.18 s for MOLLI; 0.27 s for 3D VFA; and 0.50 s for 2D VFA; and for 1.5 T, 0.14 s for MRF, 0.24 s for MOLLI; 0.25 s for 3D VFA; and 0.50 s for 2D VFA. We note that this measurement is biased due to the higher numbers of low-value T_1_ vials, which increases their importance or weighting in this calculation; when low-value T_1_ vials (< 100 ms) were excluded, this mean of absolute differences between MRF and FSE-IR was 0.05 s at 3.0 T and 0.03 s at 1.5 T, while other methods were greater than 0.15 s.

For T_2_ mapping, both multi spin echo (MSE) and MRF agreed up until 300 ms, at which point the values diverged. This is accounted for by not obtaining a sufficient number of lengthy TEs during the MSE acquisition.

## Discussion

This study demonstrates the feasibility of free-breathing, non-gated MRF in the pancreas at 1.5 and 3 T within a clinically reasonable acquisition period of 2.4–3.6 min. The MRF framework allows qualitative and quantitative data to be acquired simultaneously, allowing ready comparison between longitudinal time points and against population-derived norms, as well as giving improved imaging repeatability and more meaningful interpretation of intensity changes. The total acquisition time was very low when compared with other reported single parameter acquisitions (non-MRF based), which include quantitative T_2_ measurements of the pancreas at 1.5 T: 8 min^16^ and 3 T: 2 min 50 s^9^. Similar to Wang^35^, T_1_ maps with MRF were obtained in 10 s/slice, although we did not perform breath-holding and obtained images at nearly half the voxel volume. Furthermore, the repeatable^20^ quantitative nature of MRF data has the potential to improve comparability between centres.

Despite the overall lack of motion sensitivity in MRF^21^, we found coronal acquisition to be preferred over axial. The axial artefacts were found to be due to motion rather than FOV limitation. Prior MRF studies^25^ do not report such limitations in axial images, likely because different MRF parameters were applied, leading to greater SNR at the cost of increased acquisition time. The respiration artefacts were limited in the coronal plane as the motion remained in-plane through all excitations. In the axial plane motion occurs in the slice direction, leading to underexcitation of through-plane voxels and consequently incorrect T_1_ and T_2_ values. Motion occurred occasionally in coronal acquisitions resulting in inaccurate T_1_ and T_2_ values. We found that air in the gut lead to higher field non-uniformities in T_2_ maps.

Here we report MRF-derived quantitative differences between normal tissue types, their T_1_ and T_2_ relaxation values and the relationship of these values with field strength. Similar T_1_ and T_2_ trends between organs were observed at both field strengths, although the trends were more pronounced for both T_1_ and T_2_ at 1.5 T and for T_1_ at 3.0 T. As per previous literature^34^, there was no significant difference (*p* > 0.05) in the T_1_ or T_2_ relaxation values of liver and pancreas, at both field strengths. The pancreas had lower T_1_ and T_2_ values than muscle, spleen, and kidneys at both field strengths. The mean T_2_ values of the pancreas were nearly 40% lower than those of muscle, spleen and kidney at 1.5 T. This suggests that MRF-derived T_2_ maps at this field strength can be used to discern these organs more easily than at 3 T, where the mean T_2_ values varied by < 12%.

Pancreatic processes, including cancer, have long T_1_ values (Table 2), which can be challenging to map with most techniques, as they result in lower signal recovery between pulses and require longer recovery periods for full signal relaxation. However, MRF has been reported to measure long T_1_ values accurately^20,26^, while remaining time-efficient. This study shows the ability of MRF to accurately acquire T_1_ and T_2_ maps of the normal pancreas, despite its relatively long T_1_ and T_2_ values. The signal homogeneity throughout the normal pancreas in both T_1_ and T_2_ maps at 1.5 and 3.0 T was a key observation, as it strengthens the use of MRF in the context of pancreatic disease. This will potentially allow depiction of regional variability/heterogeneity, and definition of boundaries between pancreatic processes and normal pancreatic tissue within the same patient, which would have clear clinical impact, particularly in the context of PCa. Clinical overlap between PCa and chronic pancreatitis (CP) is well recognised, as CP increases the risk of PCa and often coexist with PCa^33,36^. We envisage, that distinguishing the two processes by MRF might prove challenging, but further studies with histopathological correlation will be needed.

Despite the higher T_1_ values at 3.0 T (1010–1041 ms) obtained in this study, these remained 300–700 ms lower than those measured in patients with pancreatic disease using MOLLI (1324 ms for CP; 1675 ms for pancreatic ductal adenocarcinoma)^35^, suggesting that MRF would still be able to distinguish normal pancreatic tissue from diseased pancreas. However, we envisage that if MRF in coronal plane was to be used, then disease pancreas would also have proportionally higher values. Ascites is often present in patients with advanced PCa. The presence of large volume of ascites impacts on image quality, as it creates B1 field non-uniformity. However, previous studies from our group have shown that high quality maps could be obtained in patients with large ascites^26^.

The MRF-T_1_ values at 1.5 T and 3.0 T were shown to be 27% (584 ms) and 15% (865–884 ms) higher than in the literature, where MOLLI was used for in vivo measurements, respectively^10,14,34,35^. However, MOLLI can be affected by variable heart rates, incomplete tissue recovery between inversion pulses, and adiabatic inversion inefficiencies resulting in an underestimation of T_1_ by as much as 25% at 3.0 T^37^ and 17% at 1.5 T^38^. Similarly to our results, T_1_ miscalculation has been reported with cardiac MRF, where the MRF-T_1_ values were 173 ms higher than MOLLI-T_1_ values^36^ and 97–189 ms lower than SASHA-T_1_ values^39,40^. Paradoxically, our phantom work demonstrated lower MRF-T_1_ values than MOLLI-T_1_ values, indicating the complexity and multifactorial nature behind the in vivo measurements. Our MRF-T_1_ results were also ~ 200 ms longer than those reported by prior MRF studies^15,25^, which more closely matches MOLLI values. We found that this difference was likely due to a combination of technical and biological factors, as we included different MRF parameters, acquisition plane and a younger cohort. Coronal acquisition was found to increase T_1_ values when compared with axial acquisition (used in the other studies). The significantly younger mean age of our cohort might have contributed to a lesser extent to our results. The MRF T_2_ relaxation times, at both field strengths, were also in the higher range, which could be due to factors inherent to the sequence^9^.

In this study, we also found that organs lying at the image periphery, such as muscle, kidney and spleen, showed a wide T_1_ and T_2_ SD values and higher T_1_ mean differences between acquisition plane, independently of the field strength, likely due to MRF inherent k-space undersampling at the edges causing motion-like artefacts and poor dictionary matching. These artefacts could be reduced by increasing voxel size or the number of frames such that the SNR is increased at the cost of increased time acquisition. Also, the T_1_ and T_2_ values showed multi-modal distribution for both field strengths and tissue type, which was partially caused by the few number of participants (n = 16). The distributions for each organ did not simply shift between field strengths, but resulted in non-trivial transformations, likely due to slightly different analysed anatomic locations as an effect of patient position/respiration. Despite these limitations, MRF is one of few methods that can obtain free-breathing T_1_ and T_2_ parameter maps within reasonable acquisition times.

This work was also challenging due to computational limitations involving the high dimensionality of the acquired and simulated datasets, which limited either acquired raw data or matching of transient state simulation parameters. The approximately 4 min abdominal MRF scan used approximately 4 gigabytes of disk space due to near continuous data acquisition combined with the large number of coil channels. The original MRF paper^19^ used 32 coil channels, a matrix size of 128 × 128, a single slice, and 1000 frames, whereas our work uses near 20 slices with twice the matrix sizes. Reconstruction prior to dictionary matching required memory reduction steps, such as coil combination and SVD compression. During dictionary matching where the inner product was calculated between the simulated dictionary and compressed acquisition data, the maximum amount of RAM used was 350 gigabytes while using 44 threads (Xeon Gold 6152). When B_1_^+^ or B_0_ values were simulated, the T_1_ and T_2_ maps appeared much noisier, and therefore these were not performed.

This proof-of-principle study has shown the feasibility of using free-breathing, non-gated coronal MRF for fast imaging and quantification of relaxation parameters in the normal pancreas. We envisage that the MRF framework will be of great value in patients with PCa, who are usually frail and with limited tolerance to long examinations or breath-hold MRI measurements. The MRF technique might also prove useful in characterising and grading pathological conditions such as CP, given its ability to acquire simultaneous mapping of T_1_ and T_2_ as well as qualitative images. Furthermore, the demonstrated feasibility of MRF in the abdomen at 1.5 T could significantly impact on the clinical potential of MRF as an imaging tool, as 1.5 T remains the most widely used field strength worldwide.

## Materials and methods

Sixteen healthy volunteers were imaged in the supine position with free-breathing MRF using a 32-channel abdominal array on a 3.0 T MRI system, after informed consent, with twelve of the sixteen also imaged on a 1.5 T MRI system (MR750 and MR450, GE Healthcare, Waukesha, WI, USA, respectively). Fasting was not requested to the volunteers.

The present study protocol was reviewed and approved by the Hertfordshire Research Ethics Committee (REC ref 08/H0311/117, IRAS 161555, REC approval on 12 Sept 2008). The present study was performed in accordance with relevant guidelines and regulations. To protect the individuals’ privacy, the patient’s exam information was pseudo-anonymised by replacing personal identifiers with pseudonyms. All work was carried out in accordance with relevant guidelines and regulations.

### Phantom protocol

**Figure 7:**
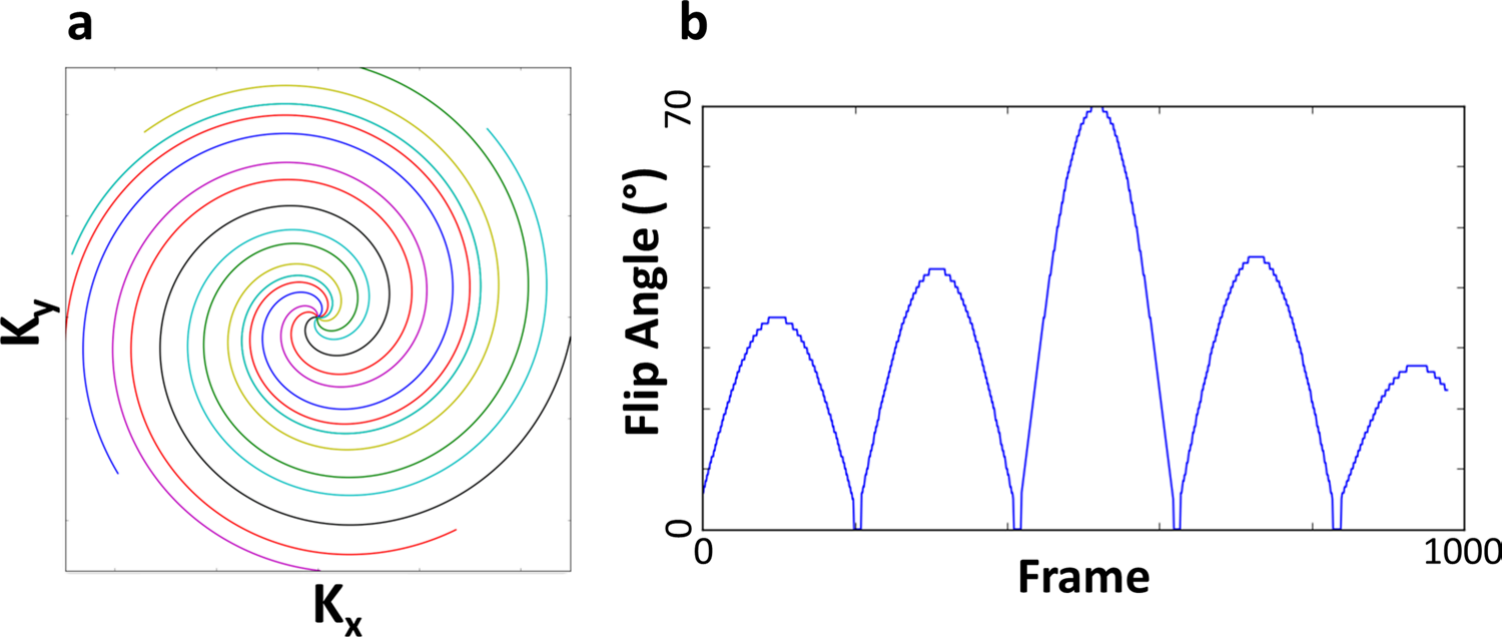
(**a**) The acquisition consisted of undersampled spirals that were rotated by the gold-angle after each TR. The first nine spirals are shown here. (**b**) One spiral (or frame) was acquired per TR, with the flip angle varied per TR as shown here.

MRF data were acquired with an inversion-prepared 2D steady-state-free-precession (SSFP) MRF sequence (1, 2). The acquisition consisted of 979 undersampled interleaved spirals with 656 points per spiral, and with sequential spirals rotated by the golden-angle (Fig. 7a). The maximum gradient strength per spiral was 28 mT/m and the maximum slew rate was 108 T/m/s. The imaging parameters were: field-of-view (FOV) = 260 × 260 mm^2^, matrix = 256 × 256, slices = 3, slice thickness = 3.0 mm, spacing 1.0 mm, sampling bandwidth =  ± 250 kHz, slice dephasing = 8π, echo time (TE) = 2.5 ms, repetition time (TR) = 10 ms, acquisition time = 9.79 s/slice. The flip angle lists matched those in Jiang et al.^41^ (Fig. 7b). A static TR was used as the random TRs listed in Jiang et al.^41^ gave an unpleasant auditory pitch.

For VFA-T_1_ mapping, both 2D and 3D data were acquired with a fast spoiled gradient echo (FSPGR) method using flip angles of 2, 5, 8, 12, 15, 18, 22, and 26°. The 2D data matched the FOV, matrix, and slices as the MRF acquisition. For FSE-IR-T_1_ mapping, data were acquired with inversion times (TIs) of 50, 100, 200, 400, 800, 1600, and 2400 ms. T_1_ data for all non-MRF techniques were fit using non-linear fitting of the signal equations.

Multi spin echo (MSE) data for T_2_ estimations were acquired with TEs = 8.1, 16.3, 24.4, 32.6, 40.7, 58.9, 57.0, 65.2 ms. MSE data were fit with a log linear least squares algorithm.

### In vivo protocol

MRF data were acquired with the same parameters as in the phantom. Coronal and axial images were acquired at 3.0 T, but only coronal images were acquired at 1.5 T due to fewer respiratory artefacts than axial images.

Images were also obtained with a coronal single-shot-fast-spin-echo sequence at 3.0 T during a 33 s breath-hold with TR = 1132 ms, TE = 80 ms, matrix = 448 × 224, field-of-view = 360 × 324 mm^2^, slice thickness = 6 mm, slices = 24, bandwidth =  ± 83.33 kHz, coil acceleration factor = 2. At 1.5 T, with TR = 1102, TE = 62 ms, matrix = 320 × 224, FOV = 460 × 460 mm^2^, slice thickness = 5 mm, slices = 26, and bandwidth =  ± 83.33 kHz.

### MRF Image Reconstruction

Each under-sampled spiral was reconstructed to give 979 under-sampled images per slice. The spiral k-space was regridded and interpolated to a Cartesian k-space before a Fast Fourier Transfer (FFT), and used a three frame sliding window^42^. The images were reconstructed with 48 parallel CPUs and used 400 gigabytes of RAM. After reconstruction, each coil channel was combined using adaptive coil combination based on weights determined from the average of the time frames^43^. The undersampled images were reduced from 979 to 16 images using the SVD decomposition weights determined during dictionary compression^44^.

### MRF dictionary simulation

Dictionary simulations of the signal evolution in a steady-state-free precession acquisition scheme were performed using the extended phase graph formalism^45^. The slice profile was also included. The ranges and incremental (step-size) changes of the T_1_ and T_2_ values that were simulated in the dictionary were T_1_ = [0.01:0.005:1;1:0.04:6] seconds ([minimum: step-size: maximum]), and for T_2_ = [0.005:0.001:0.1; 0.1:0.01:4; 4:0.04:6] seconds (where the semi-colons indicate concatenated lists). The dictionary size was compressed to 16 singular vectors (rank) with SVD to reduce the size for long term storage and faster dictionary matching^44^.

### MRF pattern matching

MRF uses a pattern recognition algorithm to identify the T_1_ and T_2_ tissue properties in each voxel. The T_1_ and T_2_ maps from MRF were obtained by inner product pattern matching of the dictionary, which is a signal look-up table based on simulations with different T_1_ and T_2_ times, with the best match to the acquired reconstructed data.

The inner products between the normalized measured signal evolution of each voxel and each normalised dictionary entry are calculated. The dictionary entry returning the maximum value for the inner product is taken as the best representation of the acquired signal evolution. The respective T_1_ and T_2_ values are consequently assigned to the voxel. The rPD (relative proton density) is calculated as the scaling factor used to match the dictionary simulation with the measured signal evolution.

### Region-of-interest selection

Pancreatic (head, body and tail), liver (right hepatic lobe avoiding the inclusion of vessels), kidney (most cases lower pole, including cortex and medulla), spleen and muscle (right psoas muscle) sub-regions (regions of interest) were identified and drawn manually in both T_1_ and T_2_ MRF maps, for each subject and imaging exam, via tracing with a computer mouse by a trained reader using custom in-house software.

### Statistical analysis

Data were analyzed using GraphPad Prism v6 (GraphPad Software, San Diego, USA). Data were reported as mean ± SD, unless stated otherwise. Statistical significance was tested with Prism using unpaired Student's *t*‐test and ANOVA tests to compare quantitative parameters between groups. The results were considered to be significant when *p* < 0.05.
